# Gene set of chemosensory receptors in the polyembryonic endoparasitoid *Macrocentrus cingulum*

**DOI:** 10.1038/srep24078

**Published:** 2016-04-19

**Authors:** Tofael Ahmed, Tiantao Zhang, Zhenying Wang, Kanglai He, Shuxiong Bai

**Affiliations:** 1 State Key Laboratory for the Biology of the Plant Diseases and Insect Pests, Institute of Plant Protection, Chinese Academy of Agricultural Sciences, Beijing 100193, China; 2 Bangladesh Sugarcrop Research Institute, Ishurdi-6620, Pabna, Bangladesh

## Abstract

Insects are extremely successful animals whose odor perception is very prominent due to their sophisticated olfactory system. The main chemosensory organ, antennae play a critical role in detecting odor in ambient environment before initiating appropriate behavioral responses. The antennal chemosensory receptor genes families have been suggested to be involved in olfactory signal transduction pathway as a sensory neuron response. The *Macrocentrus cingulum* is deployed successfully as a biological control agent for corn pest insects from the Lepidopteran genus *Ostrinia*. In this research, we assembled antennal transcriptomes of *M. cingulum* by using next generation sequencing to identify the major chemosensory receptors gene families. In total, 112 olfactory receptors candidates (79 odorant receptors, 20 gustatory receptors, and 13 ionotropic receptors) have been identified from the male and female antennal transcriptome. The sequences of all of these transcripts were confirmed by RT-PCR, and direct DNA sequencing. Expression profiles of gustatory receptors in olfactory and non-olfactory tissues were measured by RT-qPCR. The sex-specific and sex-biased chemoreceptors expression patterns suggested that they may have important functions in sense detection which behaviorally relevant to odor molecules. This reported result provides a comprehensive resource of the foundation in semiochemicals driven behaviors at molecular level in polyembryonic endoparasitoid.

Olfaction is a finely tuned sense of smell. It plays critical role to detect chemicals cues in their environment, essential for insects in foraging, host-seeking, mating choice, oviposition site locating by females, warning and defense^1^,^2^,^3^. Accurate detection of volatile compounds in the surrounding environment is required for their survival.

In almost all of the insects, repertoires of several ones to several hundred highly divergent odorant receptors (ORs) are responsible for detecting a myriad of volatile chemical signals in the environment^4^. Insect chemoreceptors consist of three major gene families appear to form binding sites for odorant molecules at the membrane surface of olfactory sensory neurons (OSNs)^5^,^6^,^7^, the odorant receptors (ORs)^8^,^9^,^10^,^11^, gustatory receptors (GRs)^12^,^13^,^14^,^15^,^16^,^17^, and recently discovered variant ionotropic glutamate receptors (IRs)^5^,^18^,^19^, whose environmental chemical signals are converted into electrical signals interpreted by the nervous system – starting with the binding of odor molecules to odorant receptors neuron (ORN) dendrites to the brain^20^,^21^.

The ORs of both insects and vertebrates have seven transmembrane domains (TMDs), but insects ORs do not belong to the canonical G-protein coupled receptors (GPCRs), to which have a reversed membrane topology (intracellular N-terminus)^8^,^22^. The first ORs genes, identified in the genomic analysis of *Drosophila melanogaster* encoded seven transmembrane domains that were largely expressed in morphological and functional types of olfactory sensilla, especially trichoid and basiconic sensilla^8^,^10^,^11^. Multiple ORs have now been identified in species from at least four insect orders, including Diptera, Lepidoptera, Hymenoptera and Coleoptera^23^,^24^,^25^,^26^,^27^. These ORs display a high divergence (only 20–40% identities) in their sequences among or within species^28^. Insect ORs are frequently co-expressed with a nonconventional OR previously referred to as OR83b in *D. melanogaster*, OR2 in *Bombyx mori*, and OR7 in mosquitoes, but these nonconventional OR have been universally named as olfactory receptor co-receptor (Orco)^29^. The ORs detect a variety of odor compounds^30^,^31^, including pheromones^32^ and microbe derivative or plant volatile compounds^33^. Some of ORs are characterized by their response specificity^33^, whereas others appear more broadly tuned at high stimulus concentrations^30^.

GRs are mostly expressed in gustatory receptor neurons in taste organs involving in contact chemoreception^34^. Insect GRs and ORs are distantly related members of the same superfamily^9^. The GRs are more conserved in sequence and structure than ORs^35^,^36^, probably due to a comparatively smaller search space on associated cues. These GRs typically detect sugars, bitter compounds, and contact pheromones^37^.

IRs were discovered in *D. melanogaster* by bioinformatic analyses as another class of receptors involved in chemoreception^5^. Apparently, IRs are related to ionotropic glutamate receptors (iGlurs), which are involved in synaptic signal transduction in both vertebrates and invertebrates^20^,^34^. Unlike ORs, IRs have been identified throughout protostomia (including arthropods, mollusks, annelids and nematodes) and, thus, constitute a far more ancient group of receptors than the ORs^19^. Due to the IRs have atypical binding domains that are more conserved than ORs, it is possible to identify several paralogous lineages among insects^20^. IR-induced responses appear to be conferred by assemblies of variable subunits in a heteromeric receptor, as up to five different IRs can be co-expressed in a single OSN^5^. A functional complex formed by two or more subunits of IRs, including odor-specific receptors and one or two broadly expressed receptors (in *D. melanogaster*, IR25a and IR8a) that function as co-receptors^38^. IRs in insects are divided into major two types: the “antennal IRs” are conserved across insect orders with chemosensory function, and the “divergent IRs” is species-specific and assigned a tentative role in taste^19^.

Except the receptor genes, several multigene protein families have also been discovered to play critical roles in olfaction, including: odorant binding proteins (OBPs)^39^,^40^; chemosensory proteins (CSPs)^41^,^42^; sensory neuron membrane proteins (SNMPs)^43^,^44^; and odorant degrading enzymes (ODEs)^45^. Several extensive studies have been described the characteristics and potential roles of these genes in insect olfaction^5^,^37^,^42^,^43^,^45^,^46^.

Identification of receptors gene families has largely been possible in insects of which genomic data are available due to their abundance and sequence diversity^17^,^47^. With the recent advances in RNA-Seq and computational technologies has been used widely such type identifications in non-model organisms. Usages these technologies, a wide range of insects olfactory genes have identified and reported of which no sequenced genome is available^20^,^34^,^47^,^48^,^49^,^50^,^51^. Within the *Macrocentrus cingulum* Brischke (Hymenoptera: Braconidae), very limited number of olfactory genes including co-receptors^52^ have been identified. However, only one candidate gene from OBP family (McinOBP 1) has been identified with function study^1^, while others candidate genes remaining to be identified.

The identification of olfactory receptors genes families- ORs, GRs and IRs in polyembryonic endoparasitoid wasps will provide information regarding their chemical communication and it’s crucial for genetic manipulation of their sensitivity to chemical cues using in biocontrol systems. This research investigated the antennal chemosensory gene families of the *M. cingulum* by antennal transcriptomes from next-generation sequencing. *M. cingulum* is a polyembryonic endoparasitoid of the Asian corn borer, *Ostrinia furnacalis* (Guenée) (Lepidoptera: Crambidae) and the European corn borer, *O. nubilalis* (Hübner)^53^,^54^ and is distributed across Europe and throughout Asia, including Japan, Korea and China^55^. *M. cingulum* has evolved an efficient olfaction system like other parasitoids use herbivore induced plant volatiles (HIPVs) and green leaf volatiles (GLVs) from infested plants by host insect as chemical cues. Female *M. cingulum* use host larval frass in stalk tunnels as host-searching cues^56^. Our previous study with this wasp, only 9 ORs has been identified (accession number KC887063-71), consequently, additional receptors genes to be investigated to permits a better understanding of the molecular basis of polyembryonic endoparasitoid olfaction. Identification of the chemosensory genes can ultimately lead to comprehensive understanding of how to recognize and locate host for oviposition in this wasp. Our present research investigated the antennal chemosensory gene families of the *M. cingulum* from antennal transcriptomes by next-generation sequencing.

## Results

### Sequencing and assembly

Through the Illumina HiSeq 2500 RNA-Seq strategy, the male and female *M. cingulum* antennal transcriptomes generated of 2,73,13,634 and 2,64,69,263 clean reads respectively, and assembled to 57,179 transcripts and 41,254 unigenes with 1,571 bp and 982 bp mean length respectively (Table 1). The sample GC content was 41.22% for female and 39.98% for male, and the average quality value was ≥30 for female 90.49% and 90.41% for male (Table 2). The N50 was 3,787 bp for transcripts and 2,383 for unigenes. Approximately 5,353 unigenes were longer than 2 kb. The total length of the assembled transcriptome was about 40.5 Mbp. The clean reads of the *M. cingulum* antennal transcriptome were deposited in the NCBI_SRA database, under the accession number of SRR2968845.

### Functional annotation

**Figure 1:**
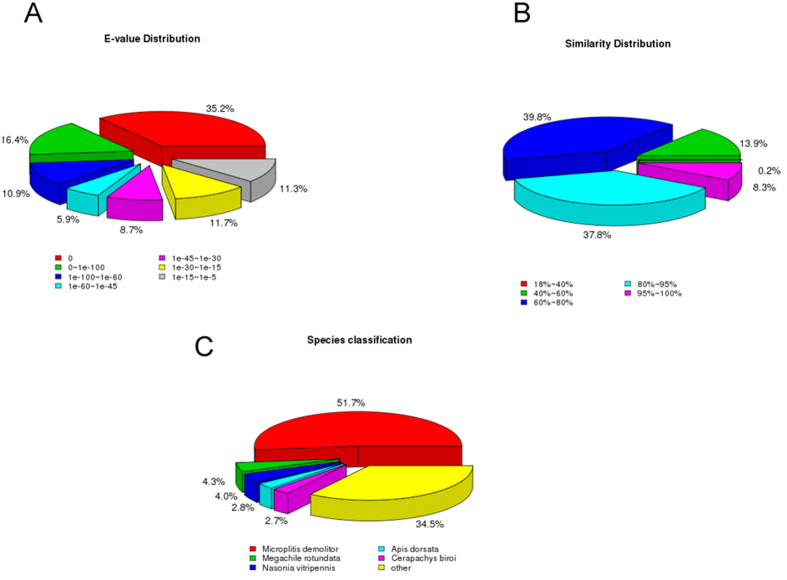
Homology analyses results. The BLASTx annotations of *M. cingulum* antenna transcripts (**A**) *E*-value distribution, (**B**) similarity distribution, and (**C**) species distribution.

The functional annotations of the unigenes were performed mainly with deposited in diverse protein datasets listed in methods section. In total of 41,254 unigenes, 10,977 (26.6%) unigenes in the Nr database, 4,283 (10.38%) unigenes Nt data base, 5,248 (12.72%) in the KO database, 8,737 (21.17%) unigenes in the SwissPort database, 10,741 (26.03%) unigenes in the Pfam database, 10,781 (26.13%) unigenes in the GO database, 7,110 (17.23%) in the KOG database, 2,197 (5.32%) in the all database and 14,113 (34.21%) unigenes in the at least one database were annotated (Table 3). Using Nr database annotation, 7,114 (64.8%) unigenes matched to known proteins. Among the 10,977 annotated unigenes, 5,313 (48.4%) had a strong match (*e*-value smaller than1e^−45^) whereas 1,240 (11.3%) showed poor homology with *e*-value between 1e^−15^ and1e^−5^ (Fig. 1A). From the Nr annotation, 39.8% unigenes showed 60–80% and 37.8% unigenes showed 80–95% similarity with known proteins (Fig. 1B). Nr database queries revealed that 65.5% sequences closely matched to hymenopteran sequence (*Microplitis demolitor* 51.7%, *Nasonia vitripennis* 4.0%, *Apis dorsata* 2.8%, *Cerapachys biroi* 2.7 and *Megachile rotundata* 4.3%) (Fig. 1C).

**Figure 2:**
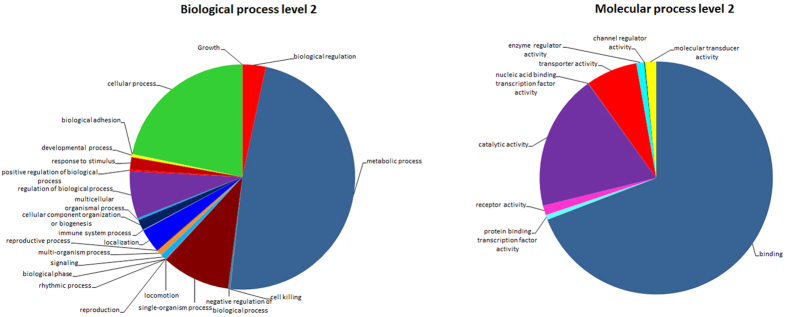
Gene ontology (GO) annotation summary. GO analysis corresponding to 10,781 contig sequences in *M. cingulum*, as predicted for their involvement in (**A**) molecular function (level 2 GO categorization) and (**B**) biological process (level 2). For results presented as detailed bar diagrams, see supplementary Fig. S1.

From Gene Ontology (GO) annotation the *M. cingulum* antennal transcriptome unigenes (10,781 of 41,254 unignes) were associated with GO terms which cover three domains: biological process, cellular component and molecular function (Fig. 2, and supplementary Fig. S1). In the terms of biological process; cellular, metabolic and single organism processes represented most of genes. In the molecular function terms, mostly associated with binding activity (e.g. nucleotide binding, odorant binding, ion binding), and catalytic activity (e.g. hydrolase and oxidoreductase activity). In the cellular category, cell, and cell part were the most abundant (Fig. 2).

**Figure 3:**
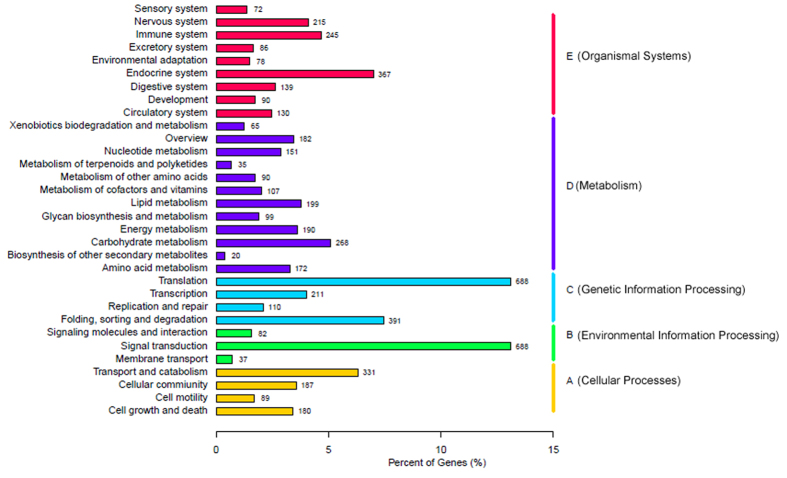
Kyoto Encyclopedia of Genes and Genomes (KEGG) annotation summary. KEGG distribution of the *M. cingulum* unigenes were annotated by 256 pathways in 5 major groups.

In total 5,994 unigene sequences are annotated to 256 pathways using with Kyoto Encyclopedia of Genes and Genomes (KEGG) pathway database to identify the metabolic pathways which are populated by these unigenes. The “metabolic pathway” populated with highest number of unigenes (1,578, 26.33%) followed by Cellular Processes pathways (1,422, 23.72%); and “Organismal Systems pathways” (1,400, 23.35%) (Fig. 3). This annotation information helps to conduct further research on metabolic function and pathways, and biological behaviors of *M. cingulum* genes.

### Identification of olfactory receptor gene families

#### Odorant receptors

Bioinformatic analysis of the *M. cingulum* antennal transcriptomes identified 109 sequences including previously described ORs McinOR1-9 and *McinOrco*^52^ that encode candidate OR genes. The transcript name, length, best BLASTx hit, identity, and male or female specificity was summarized in Table 4. While the length of 20 other amino acid sequences ≤100 are provided as supplementary material in Table S2. A full-length *McinOrco* gene coding 479 amino acids was easily identified because it contained the intact open reading frame and seven transmembrane domains, which are typical characteristics of insect ORs. The majority of partial length transcripts possess overlapping regions with low amino acid sequence identity, indicating that they represented separate individual proteins. However, the possibility that the remaining non-overlapping transcripts represented fragments of individual proteins cannot be excluded; therefore, the total number of McinORs reported could be reduced by 20, based on sequence alignments and subsequent fragment location (i.e. C-terminus, internal, or N-terminus). We eventually analyzed 89 OR (including our previous identified ORs/Orco) sequences in our phylogenetic analysis.

**Figure 4:**
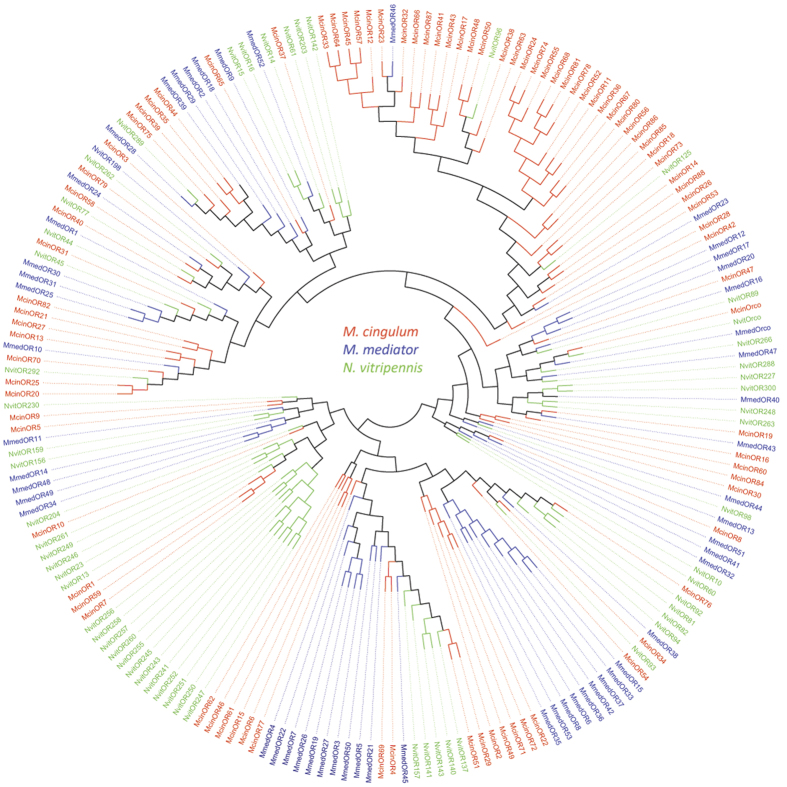
Phylogenetic tree of odorant receptors (ORs). Included are ORs from *M. cingulum* (Mcin), *M. mediator* (Mmed), and *N. vitripennis* (Nvit).

With exception of Orco, the predicted ORs shared quite low identity probably due to the high variance among OR gene family. Only three of 79 ORs (McinOR12, McinOR15, and McinOR42) showed more than 50% identity with known ORs in NCBI database (Table 4). The phylogenetic analysis also showed that ORs were extremely divergent between species but formed monophyletic group within same species (Fig. 4). However the highly conserved Orco shared 95–99% amino acid sequence identity and clustered in same branch with orthologous relation among three species (Fig. 4). All of the other McinORs were distributed in different branches of the phylogenetic tree. Eight species specific subgroups were identified consisting of different numbers of McinORs. The highest 14 McinORs (OR11, 24, 36, 52, 55, 56, 67, 68, 74, 78, 80, 81, 85, and 86) and the lowest three McinORs (OR30, 60 and 84) and another three McinORs (OR1, 7 and 59) clustered within the species specific subgroup. Others McinOR are clustered with *N. vitripennis* or *M. mediator* ORs.

#### Gustatory receptors

**Figure 5:**
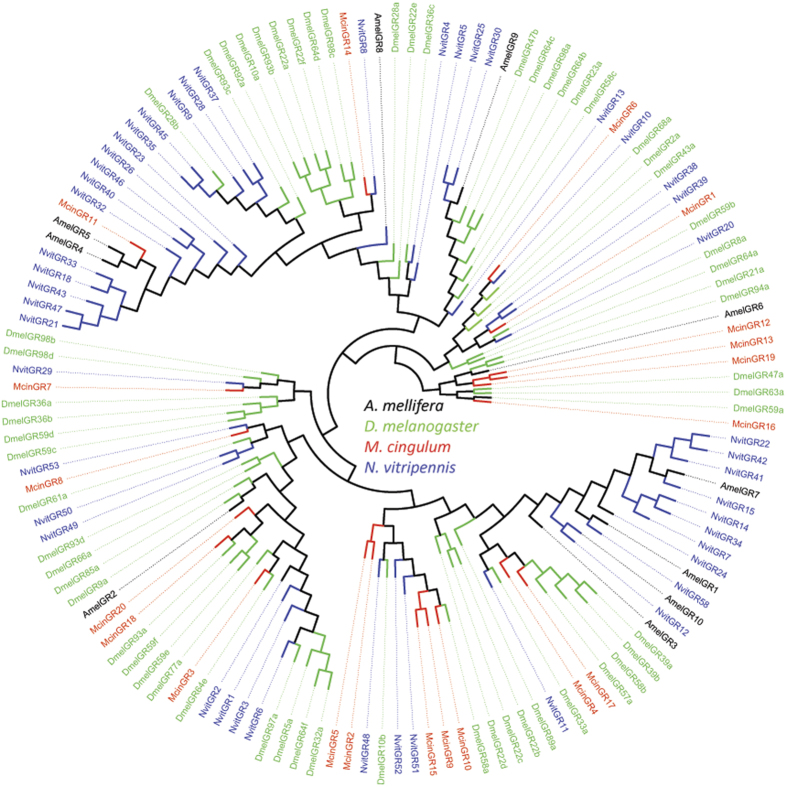
Phylogenetic tree of gustatory receptors (GRs). Included are candidate *M. cingulum* GRs with *A. mellifera* (Amel), *D. melanogaster* (Dmel) and *N. vitripennis* (Nvit) GRs.

We identified 20 transcripts encoding candidate GRs in the *M. cingulum* antennal transcriptome (Table 5). Most of candidate McinGRs were partial transcripts (only six represents full length protein), encoding overlapping but distinct sequences. This shows individual genes though it’s being fragment of protein sequence. A phylogeny was built with these 20 McinGRs, *N. vitripennis, A. mellifera* and *D. melanogaster* (Fig. 5). Based on the phylogenetic analysis, McinGRs were also observed to group with their presumed *Drosophila* orthologues, which have been shown to have roles in carbon dioxide detection (GR21a and GR63a)^57^,^58^ and are members of the candidate sugar GR64 receptor subfamily (GR64e)^59^ or bitter (DmelGR93a)^60^ *Drosophila* receptors (Fig. 5).

#### Ionotropic receptors

**Figure 6:**
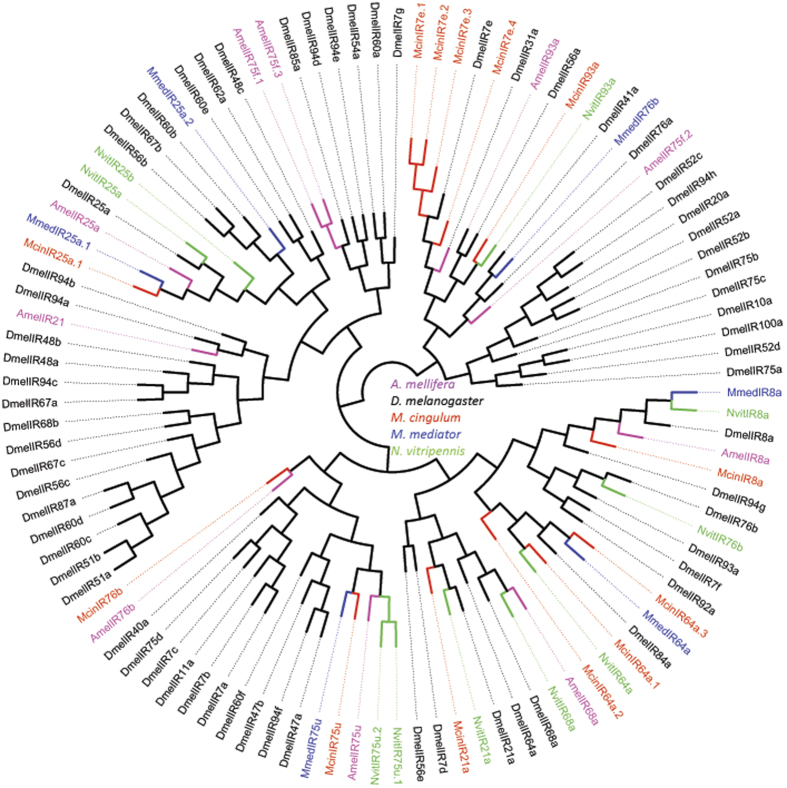
Phylogenetic tree based on protein sequences of ionotropic receptors (IRs). Included are IRs from *M. cingulum, M. mediator* (Mmed), *A. mellifera* (Amel), *D. melanogaster* (Dmel) and *N. vitripennis* (Nvit).

We identified 13 transcripts for putative ionotropic receptors in *M. cingulum* antennal transcriptome according to their similarity to IR sequence of other insects. Comparative analysis revealed that one candidate IR (MmedIR8a) was deemed as IR8a homolog to its high identity (71%). IR25a and IR76b shared 57% and 46% identity with MmedIR25a.1 and MmedIR76b, respectively. It has been reported that, the above three genes (IR8a, IR25a and IR76b) are thought to play function as IR co-receptors^5^,^38^. In the phylogenetic tree of IRs, all McinIR candidates clustered with their ionotropic receptor orthologs into separate sub-clades (Fig. 6). Because of the relative high conservation of IRs, all the splits of McinIRs were strongly supported by high support values. The candidate IR unigenes were named according to their similarity to known IRs. The information, including unigene reference, length, and best blastx hit of all IRs were listed in Table 6.

### Tissue and sex specific expression profile of candidate *M. cingulum* chemosensory receptors

**Figure 7:**
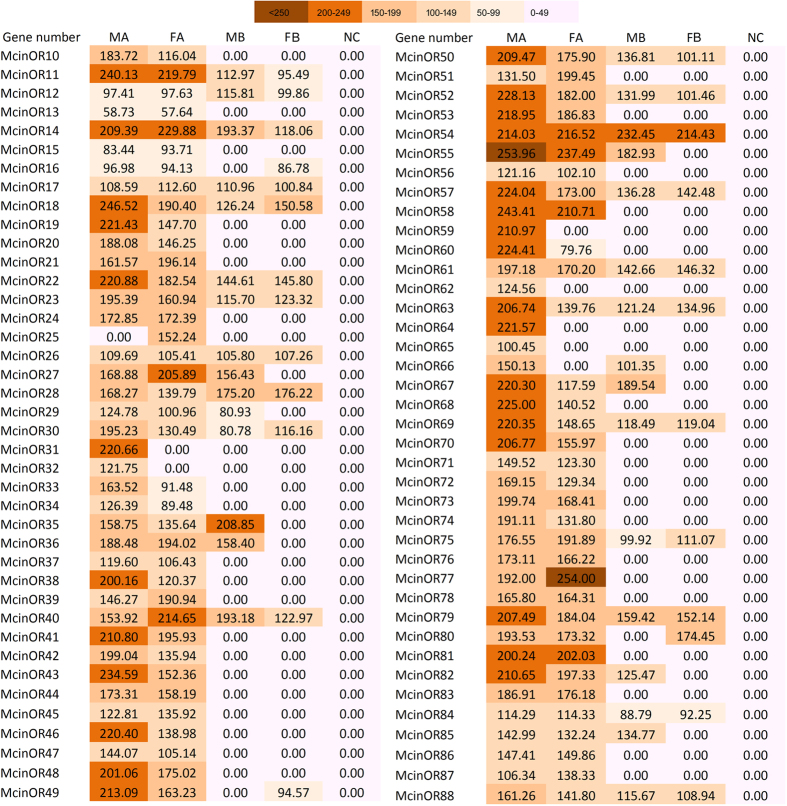
Tissue- and sex- specific expression profiles of *M. cingulum* ORs. Comparison of expression profile of olfactory receptor (OR) genes in male and female adult antennae and body as revealed by RT-PCR. In each box, the relative abundance value in (M. pixel) of each receptor gene is indicated. Color scales were established using the conditional formatting option in Excel (color scale shown inside the figure).

**Figure 8:**
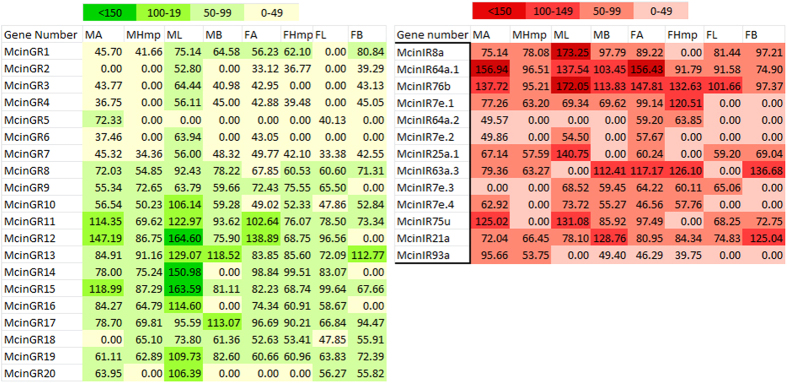
Tissue- and sex- specific expression profiles of *M. cingulum* GRs and IRs. Comparison of expression profile of gustatory receptor (GR) and ionotropic receptors (IR) genes in male and female adult antennae and head with mouth parts, leg and body as revealed by RT-PCR. In each box, the relative abundance value in (M. pixel) of each receptor gene is indicated. Color scales were established using the conditional formatting option in Excel (color scale shown inside the figure).

We performed reverse transcription PCR (RT-PCR) analyses in different tissues of adult males and females to explore the expression patterns of *M. cingulum* OR, GR and IR genes. Most of OR genes were expressed in male and female antennae, the crucial chemosensory organs, suggesting a functional role of these genes in olfaction (Fig. 7). The candidate OR31, 59, 62, 64, and 65 showed a male antenna specific expression, while only one OR25 was expressed only in female antennae. The remaining ORs were expressed in both sexes, by differential expressions in male or female among tissues. Five of them, OR11, 14, 54, 55 and 81 were most highly (>200 M. pixel) expressed in both male and female antennae (Fig. 7). Twenty-one ORs (OR18, 19, 22, 35, 38, 41, 43, 46, 48, 49, 50, 52, 53, 57, 60, 63, 67, 69, 70, 79, and 82) were clearly expressed higher in male compared to female while only two (OR27 and 40) expressed higher level in female than male. GRs and IRs showed a ubiquitous expression pattern except the McinGR12, 14 and 15 which was present predominantly in the male legs; McinIR64a prominent in male and female antennae but IR8a and 25a only in male antennae. McinIR76b dominantly expressed in male leg (Fig. 8).

**Figure 9:**
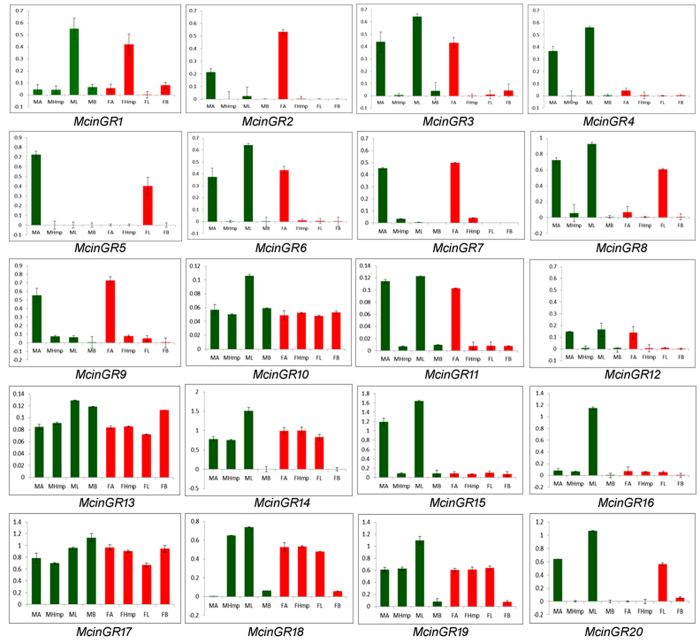
Expression patterns of McinGR genes. Transcript levels of *M. cingulum* gustatory receptors (GRs) in different tissues of adult male and females measured by qPCR. cDNAs were amplified with specific primers from antennae, heads (with mouth parts), legs, and bodies.

The quantitative real-time PCR (qPCR) was used to investigate the gustatory receptor transcript abundances in the male antennae, head with mouth parts, legs, body and female antennae, head with mouth parts, legs and body tissues. By comparing expression levels McinGR10, 13, 14, 17, 18 and 19 genes were expressed at similar level in all tested tissues except 14, 18, and 19 in body of both sexes (Fig. 9). McinGR1, 3, 4, 15 and 20 were prominently expressed only male antennae and legs where as McinGR6, 8 and 11 in male antennae, legs and female legs but 16 dominantly expressed only in male legs (Fig. 9). McinGR2, 7, and 9 were highly expressed in female antenna than male. McinGR5 were highly expressed in male antenna than female.

## Discussion

This is the first comprehensive analysis of a polyembryonic endoparasitoid antennal transcriptome for the purpose to identify the major chemosensory receptor gene families (ORs, GRs and IRs) for olfaction. The identified gene families represent a valuable genomic resource of molecular basis in *M. cingulum* due to potential target genes for manipulating parasitoid wasp’s behavior and improving biocontrol techniques.

The GO annotation demonstrated that predicted three categorize functions of *M. cingulum* transcripts overall similar as those obtained from previous reports^34^,^51^,^61^,^62^. Identified individual transcripts of olfactory gene families were also comparable with other Dipteran, Coleopteran, Hymenopteran and Lepidopteran species from those of which the antennal transcriptome has been reported^20^,^34^,^61^,^63^,^64^,^65^,^66^. The comparison of these published data sets suggested a certain level of conservation in gene expression patterns in antennae.

From the *M. cingulum* antennal transcriptome, a total of 109 OR genes were identified including with our previous studies. The total number of identified ORs in *M. cingulum* greater than the *M. mediator* (68 ORs)^51^ but less than in *A. mellifera* (170 ORs) or *N. vitripennis* (301 ORs)^67^,^68^. Identified OR genes were only from the antennal transcriptome, ORs from other tissues thus might be difficult to identify in our study. However, the differences of identified OR gene number may result from sequencing methods and depth, and/or sample preparation. The large number of ORs identified in *M. cingulum* also could result from species difference; so, further research is required for confirmation.

Insect ORs mainly expressed in the antennae^69^. Present research revealed several ORs have sex specific expression, while others showed ubiquitous expression pattern but their expression level higher in antennae. The differential expression patterns of McinORs have been supported by previous study^51^,^70^,^71^,^72^. The male antennae specific ORs (31, 59, 62, 64, 65, and 66) or male biased (18, 19, 22, 45, 38, 41, 43, 46, 48, 49, 50, 52, 53, 57, 60, 63, 67, 69, 70, 79, and 82) expression profiles may play crucial roles in the detection of sex pheromone or male specific behaviors. The female specific OR25 or female biased OR27, 40, and 58, may suggest that the female specific behaviors i.e. finding host for ovipositions or others. Additionally, ORs expressed in other organ of wasp may have physiological functions, for example a Orco expressed in the testes of *A. gambiae* was considered to involve in spermatozoa activation^71^.

The GR family of insect chemoreceptors includes receptors for sugars and bitter compounds, as well as cuticular hydrocarbons and odorants such as CO_2_. Antennal GRs of insects used for tasting purpose as well as for olfaction detects^73^. However, there are no reports of polyembryonic endoparasitoid wasp GRs in antenna. So far we know, it is the first report of GR family in this wasp chemoreceptors, although some studies described the distribution of some gustatory sensilla in wasp antennae^51^,^74^. We identified 20 putative GR-encoding transcripts from the *M. cingulum* antennal transcriptomes. The identified GRs also included potential carbon dioxide receptors, which suggested that *M. cingulum* might use CO_2_ detection as a cue for host selection, like in *B. mori*, *T. castaneum* and *mosquitoes*^21^,^75^. Orthologue GR64f clusters with sugar receptor of GR1 subfamily in Hymenoptera suggested a function of sugar detection. In addition, identified McinGRs were differentially expressed in sensory and non-sensory organs. However, the recent studies showed a wide range of non-gustatory sensory functions of insect GRs^76^, that indicated GRs probably have far more divergent functions in antennae.

Generally in insects, IRs are more conserved compared to ORs and GRs^19^, which can be categorized as divergent species-specific IRs and conserved antennal IRs^5^. The antennal IR subfamily only constituted a portion of IRs, while others belong to the divergent IRs subfamily, showed species-specific expansions that are particularly large in Diptera^19^. In *D. melanogaster*, there are 66 IRs, and 15 were antennal IRs^77^. In this study, 13 IR candidates including two co-receptors, (IR8a and IR25a) were found in antennal transcriptome. Limited number of IR genes identified in hymenoptera (only 6 in *M. mediator*, 10 in *A. melifera* and *N. vitripennis*) compared to diptera (66 in *D. melanogaster*)^19^. However, our identified IR genes close to the number of *A. melifera* and *N. vitripennis* but higher than the *M. mediator*. Sequence alignments showed that the putative McinIR8a and McinIR25a had high similarity with the MmedIR8a and MmedIR25a respectively. These two mostly expressed in female antennae than in male wasp, which probably play a significant roles in host recognition for oviposition^51^. However, the ubiquitous expression feature of McinIRs revealed that these genes may have other physiological functions in non-olfactory organs.

The *M. cingulum* transcriptome data indicated that the chemosensory gene repertoire was largely similar between the male and female, only differences in the relative levels of expression of individual ORs (Fig. 7). Therefore, while male and female antennae likely perceive similar odor stimuli, their sensitivities, and hence the odour significance to the male and female, may differ. Female *M. cingulum* uses host larval frass in combined with different volatile cues for host-searching. The herbivore induced plant volatiles (HIPVs) and green leaf volatiles (GLVs) are consider to be used by female wasps from infested plants by host insect as chemical cues^78^,^79^. The female specific or biased ORs in antennae may play important role in recognition of host volatiles, which can provide a key starting to manipulate and developed OR in wasp for finding host and used as a biological tools for pest control.

## Conclusion

This study reports the first antennal transcriptome analysis in polyembryonic endoparasitoid *M. cingulum*. The genes reported here provide valuable insight into the molecular mechanisms of olfaction of this wasp. Ultimately, a large number of ORs, GRs and IRs in *M. cingulum* are identified, however the additional molecular and functional experiments are required to confirm the expression and roles of these genes. Our results provide a foundational knowledge to explore and understand the chemosensory receptor gene families of this wasp. It is promising to conduct transcriptomic analysis via next generation RNA-Seq for non-model organisms especially for polyembryonic parasitoid.

## Methods

### Insects

*M. cingulum* were collected from *O. furnacalis* larvae, which lived on infested corn plants in Langfang Experimental Station of Institute of Plant Protection, Chinese Academy of Agricultural Sciences, China. The parasitoids emerged as mature larvae from the host larvae and pupated inside the silken cocoon. Adult parasitoid wasps were fed with 20% honey solution. A laboratory colony was established and maintained at 25 °C with a 16 hr light: 8 hr dark photoperiod on host larvae of *O. furnacalis* that were reared on an artificial diet followed published procedures^80^.

### Total RNA extraction

Antennae were cut from 1–2 days old male and female wasps following snap freezing in liquid nitrogen. The collection of head with mouth parts, legs, thoraxes, and abdomen (wingless) collected from same aged wasps were used for the RT-PCR validation of gene sequences. All the tissues were immediately stored at −80 °C for further processes. Total RNA was extracted from the antennae or other tissues using TRIzol reagent (Invitrogen, Carlsbad, CA, USA) as per manufacturer’s instructions. The RNA integrity was verified by 1% agarose gel electrophoresis and quantity was assessed with a Nanodrop ND-2000 spectrophotometer (Nano- Drop products, Wilmington, DE, USA). The integrity of the RNA was checked by the Agilent 2100 (Agilent Technologies Inc., CA, USA) before transcriptome sequencing.

### Antennal transcriptome generations

Synthesis of cDNA and Illumina library generation was completed at Novogene Co., Ltd. Beijing, China, using Illumina HiSeq2500 sequencing. The FastQC tool was used to obtain read statistics, assess read quality, and to remove the low quality data. The high-quality reads were obtained by removing adaptor sequences, empty reads low-quality sequences (reads with unknown “N” >10% sequences), and the reads with more than 50% Q ≤ 20 base on the raw reads. All the analysis based on the clean reads. The the transcriptome data was combined and *de novo* assembled using Trinity^81^,^82^. Trinity RNA-Seq is highly capable of overcoming quality and polymorphism issues due to bubble popping algorithms in each of the three modules, Inchworm, Chrysalis and Butterfly. In order to get the comprehensive information of the genes, we annotated the genes based on Nr, Nt, Pfam, KOG/COG, Swiss-prot, KEGG, GO databases. Open reading frames were predicted using ESTScan 3.0 project. Gene expression levels were estimated by RSEM software^83^, and differential expression analysis of two groups was performed using the DESeq R package (1.10.1)^84^. P-value was adjusted using q-value^85^ and q-value < 0.005 and log_2_ (fold_change) >1 was set as the threshold for significantly differential expression.

### Gene identification and annotation

For sequence homology assessment of both male and female *M. cingulum* antennal transcriptomes, gene ontology (GO) annotation was performed using Blast2Go via searches against the NCBI non-redundant protein database (using BLASTp with a 1e^−10^ threshold)^86^,^87^. GO annotated genes or transcripts were described into three domains: to molecular function, biological process, and cellular component, allowing for meta-analyses of gene populations^61^,^88^. Identification of putative chemosensory genes families by custom data base nucleotide Blast profile searches using known sequences as queries. *M. cingulum* chemosensory genes were in turn used as queries to identify additional genes (tBLASTx and BLASTp). Repetitions were completed until no new ones were identified. Identification of candidate genes was verified by additional BLAST searches using the *M. cingulum* contigs as queries against the NCBI non-redundant protein database (BLASTx). Protein domains (e.g. transmembrane domains, signal peptides, secondary structures, etc.) were predicted by queries against InterPro using the InterProScan Geneious software plugin running a batch analysis (e.g. HMMPanther, SignalPHMM, Gene3D, HMMPfam, TMHMM, HMMSmart, Superfamily, etc.)^89^. Membrane topology was assessed with Phobius^90^. In addition, we used KEGG ontology (KO) enrichment analyses to further understand their biological functions. Sequences were classified based on sequence similarity, domain structure predictions, and phylogenetic analysis.

### Phylogenetic analysis

Amino acid sequences were aligned using MAFFT^91^ and BioEdit Sequence Alignment Editor 7.1.3.0^92^ for further edit. Phylogenetic relationship was deduced by the maximum likelihood method using MEGA5^93^ with the GAMMA model for rate heterogeneity and the WAG model for substitution matrix. In addition, the rapid hill-climbing search algorithm (–f d) was used. Model optimization precision in log likelihood units for final optimization of tree topology (–e) was set at 0.0001. The tree image subsequently viewed and graphically edited in Fig Tree v1.4.0 (http://tree.bio.ed. ac.uk/software/figtree/)^94^. Phylogenetic trees were based on Hymenopteran data sets. The OR data set contained 89 amino acid sequences from *M. cingulum*, together with *N. vitripennis*^67^ *M. mediator*^51^ and *A. mellifera*^68^. The GR data set contained 20 amino acid sequences from *M. cingulum*, together with sequences from *N. vitripennis, A. mellifera* and *D. melanogaster*^67^,^68^,^95^. The IR data set contained 13 *M. cingulum* amino acid sequences with *M. mediator*^51^, *N. vitripennis, A. mellifera* and *D. melanogaster*^67^,^68^,^95^ IR sequences.

### RT-PCR analysis

To explore the expression of the ORs identified from the antennal transcriptome and compare the differential expression patterns between the sexes, RT-PCR was conducted with cDNAs prepared from the male antenna, female antenna, male and female body (including head, leg, thoraxes, abdomen) for OR genes and male and female antennae, head with maxillary palp, leg and body for GR and IR genes. Independent triplicate individual samples of total RNA were isolated from the above mentioned tissues and corresponding cDNAs were synthesized using the TranScript^®^ one-step gDNA removal and cDNA synthesis supermix (TRANSGENE Biotech, Beijing, China) following the kit manual. *β-actin* was used as reference gene (accession number- EU585777.1) and it was used to select the cDNA templates on the PCR equipment. Primers were designed manually or by Primer 5 (http://frodo.wi.mit.edu/primer5/), which was listed in supplementary material (Table S1). Individual PCR reactions were repeated three times; controls consisted of no template PCRs. The PCR conditions consisted of an initial 3-min step at 94 °C, 30 cycles of 94 °C for 30-sec, 56, 57 or 59 °C (depending on primers) for 30-sec and 72 °C for 3-min and finally 10-min step at 72 °C. Products were analyzed on a 1% agarose gel and visualized after staining with ethidium bromide. The images were recorded digitally by Dolphin-DOC (Wealtec Corp.) using 1141101 CCD-Camera, 12 V ac/dc and stored on computer. The brightness of each bands were measured from digital images by using Adobe Photoshop version CS3.

### Quantitative real-time PCR measurement

Real-time quantitative PCR (RT-qPCR) was conducted to detect the relative expression levels of McinGRs in adult male and female different tissues of *M. cingulum*. The RNA/cDNA preparation of each tissue was performed in triplicate. The gene specific primer was designed using Primer express 5.0 and listed in Supplementary Material: Table S3. The housekeeping genes *β-actin* (accession number EU585777) was used in qPCR equipment as a reference gene. The gene specific primer and *β-actin* were used to measure the Ct values of the cDNA templates to ensure the Ct values were between 22 and 25. qPCR experiments were performed using 96 well plates (Applied Biosystems, Carlsbad, CA), ABI Prism 7500 Fast Detection System (Applied Biosystems, Carlsbad, CA) and Brilliant II SYBR Green qPCR master mix (Takara). qPCR was conducted in 20 μL reactions containing 50x SYBR Premix Ex Taq 10 μL, primer (10 mM) 0.4 × 2 μL, ROX reference dye II 0.4 μL (50×), sample cDNA 1 μL, sterilized ultra-pure grade H_2_O 7.8 μL. Cycling conditions were: 95 °C for 30 sec, 40 cycles of 95 °C for 05 sec and 60 °C for 30 sec. Afterwards, the PCR products were heated to 95 °C for 15 sec, cooled to 60 °C for 1 min and heated to 95 °C for 15 sec to measure the dissociation curves. No-template and no-reverse transcriptase controls were included in each experiment. To check reproducibility, each test sample was done in triplicate technical replicates and three biological replicates. Relative quantification was performed by using the comparative 2^−ΔΔCT^ method^96^. All data were normalized to endogenous *β-actin* levels from the same tissue samples and the relative fold change in different tissues was calculated with the transcript level of the abdomen as calibrator. Thus, the relative fold change in different tissues was assessed by comparing the expression level of each GR in other tissues to that in the abdomen.

## Additional Information

**How to cite this article**: Ahmed, T. *et al*. Gene set of chemosensory receptors in the polyembryonic endoparasitoid *Macrocentrus cingulum*. *Sci. Rep*. **6**, 24078; doi: 10.1038/srep24078 (2016).

## Supplementary Material

Supplementary Information

## Figures and Tables

**Table 1:** Assembly summary of the *M. cingulum* transcriptome.

	Total number	Mean length	N50 length	Total Size
Transcript	57179	1571	3787	89824627
Unigene	41254	982	2343	40491607

**Table 2:** Sequencing summary of the *M. cingulum* transcriptome.

Sample	Raw reads	Clean reads	GC%	%≥Q30
♀antennae	27653962	26469263	41.22	90.49
♂antennae	29013514	27313634	39.98	95.01

**Table 3:** Functional annotation analysis summary of *M. cingulum* transcriptome.

Database	Number of unigenes	Percentage
NR_Annotation	10977	26.6
Nt_Annotation	4283	10.38
Ko_annotation	5248	12.72
Swiss-Prot_Annotation	8737	21.17
Pfam_Annotation	10741	26.03
GO_Annotation	10781	26.13
KOG_Annotation	7110	17.23
All databases_Annotation	2197	5.32
At least one database_Annotated	14113	34.21

**Table 4:** Candidate odorant receptor transcripts identified in adult male and female *M. cingulum* antennal transcriptomes.

Gene Name	Contig ID	Contig (bp)	ORF (aa)	BLASTx best hit (GenBank acsession/name/species)	E- value	% ID	F:M	
							RSq	RT-PCR
McinOR10	c592_g1	889	293	NP_001177606.1| odorant receptor 266 [*Nasonia vitripennis*]	1E–11	26	0.44	0.63
McinOR11	c1234_g1	710	195	EGI61565.1| Putative odorant receptor 13a [*Acromyrmex echinatior*]	5E–36	34	0.53	0.92
McinOR12	c2517_g1	437	143	XP_008552621.1| putative odorant receptor 85d [*Microplitis demolitor*]	7E–55	62	0.61	1.00
McinOR13	c4077_g1	1076	340	AGS43046.1|odorant receptor Or1a [*Cephus cinctus*]	1E–78	40	0.55	0.98
McinOR14	c8354_g1	1167	389	|XP_008547952.1| odorant receptor 82a-like [*Microplitis demolitor*]	8E–63	35	0.32	1.10
McinOR15	c9201_g1	582	194	XP_008547959.1| odorant receptor 13a-like [*Microplitis demolitor*]	4E–60	51	0.26	1.12
McinOR16	c9490_g1	608	165	NP_001177598.1|odorant receptor 251 [*Nasonia vitripennis*]	4E–32	32	0.63	0.97
McinOR17	c9743_g1	765	251	NP_001164395.1| odorant receptor 82 [*Nasonia vitripennis*]	1E–57	28	0.61	1.04
McinOR18	c11069_g1	614	192	XP_011148004.1| PREDICTED: putative odorant receptor 92a [*Harpegnathos saltator*]	1E–31	33	0.96	0.77
McinOR19	c11500_g1	1110	370	|XP_008547952.1|PREDICTED: odorant receptor 82a-like [*Microplitis demolitor*]	2E–95	40	0.36	0.67
McinOR20	c11576_g1	891	290	XP_011331455.1| PREDICTED: odorant receptor 43a-like isoform X5 [*Cerapachys biroi*]	2E–49	33	0.24	0.78
McinOR21	c11829_g1	1173	346	AGG17942.1|olfactory receptor 10 [*Microplitis mediator*]	2E–98	39	0.83	1.21
McinOR22	c11862_g1	1126	375	NP_001164394.1|odorant receptor 81 [*Nasonia vitripennis]*	4E–70	34	0.43	0.83
McinOR23	c12194_g1	705	205	|NP_001177501.1| odorant receptor 60 [*Nasonia vitripennis*]	1E–26	32	0.34	0.82
McinOR24	c12240_g1	654	193	NP_001177643.1| odorant receptor 288 [*Nasonia vitripennis*]	2E–20	30	0.78	1.00
McinOR25	c12488_g1	1118	365	EZA45268.1| putative odorant receptor-1 [*Microplitis demolitor*]	3E–37	29	0.50	#DIV/0!
McinOR26	c12943_g1	948	154	NP_001229907.1| odorant receptor 53 *[Apis mellifera*]	3E–71	38	0.77	0.96
McinOR27	c12996_g1	994	273	NP_001229911.1| odorant receptor 58 [*Apis mellifera*]	2E–64	36	0.71	1.22
McinOR28	c13179_g1	747	173	NP_001177467.1| odorant receptor 10 [*Nasonia vitripennis*]	7E–54	38	0.56	0.83
McinOR29	c13458_g1	1148	310	NP_001177603.1|odorant receptor 260 [*Nasonia vitripennis*]	2E–44	27	0.51	0.81
McinOR30	c13476_g1	1121	373	NP_001177491.1|odorant receptor 44 [*Nasonia vitripennis*]	7E–59	33	0.21	0.67
McinOR31	c13904_g1	630	128	NP_001177550.1| odorant receptor 157 [*Nasonia vitripennis*]	3E–29	33	0.08	N/A
McinOR32	c14017_g1	529	169	NP_001177706.1| odorant receptor 198 [*Nasonia vitripennis*]	4E–16	29	0.10	N/A
McinOR33	c14398_g1	415	129	AGG17943.1| olfactory receptor 9 [*Microplitis mediator*]	1E–22	39	0.18	0.56
McinOR34	c14712_g1	782	260	NP_001164395.1| odorant receptor 82 [*Nasonia vitripennis*]	9E–20	26	0.46	0.71
McinOR35	c15245_g1 (2)	1068	224	NP_001177534.1| odorant receptor 125 [*Nasonia vitripennis*]	8E–24	24	0.51	0.85
McinOR36	c15542_g1	1158	378	NP_001177601.1| odorant receptor 256 [*Nasonia vitripennis*]	2E–57	32	0.55	1.03
McinOR37	c15710_g1	713	121	NP_001177471.1| odorant receptor 15 [*Nasonia vitripennis*]	1E–18	26	0.43	0.89
McinOR38	c15806_g1	915	276	NP_001177516.1|odorant receptor 93 [*Nasonia vitripennis*]	4E–49	29	0.35	0.60
McinOR39	c15883_g1	1206	340	AGG17938.1|olfactory receptor 5 [*Microplitis mediator*]	1E–74	33	1.53	1.31
McinOR40	c15902_g1	897	298	NP_001177708.1|odorant receptor 241 [*Nasonia vitripennis*]	1E–40	28	0.22	1.39
McinOR41	c15957_g1	1087	317	AGG17936.1| olfactory receptor 3 [*Microplitis mediator*]	4E–52	28	0.32	0.93
McinOR42	c16612_g1	1072	355	AGG17934.1|olfactory receptor 1 [*Microplitis mediator*]	6E–119	55	0.35	0.68
McinOR43	c16640_g1	1119	328	|AGG17940.1| olfactory receptor 7 [*Microplitis mediator*]	2E–56	33	0.22	0.65
McinOR44	c16673_g1	717	591	NP_001177710.1|odorant receptor 289 [*Nasonia vitripennis*]	1E–24	31	0.41	0.91
McinOR45	c16974_g2	1003	308	XP_011300122.1|PREDICTED: odorant receptor 24a-like [*Fopius arisanus*]	3E–89	44	0.35	1.11
McinOR46	c16982_g1	1092	343	AGG17946.1| olfactory receptor 13 [*Microplitis mediator*]	2E–24	26	0.24	0.63
McinOR47	c17113_g1	845	258	NP_001177587.1|odorant receptor 227 [*Nasonia vitripennis*]	7E–20	27	0.67	0.73
McinOR48	c17665_g1	1299	433	AGG17945.1| olfactory receptor 12 [*Microplitis mediator*]	5E–108	40	0.32	0.87
McinOR49	c17700_g1	779	166	CAM84006.1| olfactory receptor 8 [*Tribolium castaneum*]	4E–15	27	0.45	0.77
McinOR50	c17768_g1	886	282	NP_001177589.1| odorant receptor 230 [*Nasonia vitripennis*]	1E–16	28	0.32	0.84
McinOR51	c17907_g1	783	191	AGG17941.1| olfactory receptor 8 [*Microplitis mediator*]	1E–57	43	1.83	1.52
McinOR52	c18012_g1	744	284	XP_012223367.1|PREDICTED: putative odorant receptor 85d [*Linepithema humile*]	1E–24	26	0.35	0.80
McinOR53	c18194_g2	688	196	NP_001177515.1|odorant receptor 89 [*Nasonia vitripennis*]	6E–47	40	0.41	0.85
McinOR54	c18310_g1	1028	290	XP_008560864.1|PREDICTED: putative odorant receptor 71a [*Microplitis demolitor*]	6E–80	43	0.58	1.01
McinOR55	c18322_g1	1059	347	NP_001177545.1|odorant receptor 143 [*Nasonia vitripennis*]	9E–42	26	0.15	0.94
McinOR56	c18374_g1	1176	328	NP_001177502.1|odorant receptor 61 [*Nasonia vitripennis*]	2E–46	30	0.53	0.84
McinOR57	c18647_g1	1155	385	|NP_001177517.1|odorant receptor 94 [*Nasonia vitripennis*]	4E–47	29	0.91	0.77
McinOR58	c18685_g1	1104	352	EZA45268.1|putative odorant receptor-1 [*Microplitis demolitor*]	9E–42	29	0.46	0.87
McinOR59	c18814_g2	1093	334	NP_001229890.1|odorant receptor 10 [*Apis mellifera*]	1E–15	28	0.62	N/A
McinOR60	c19087_g1	412	370	NP_001177543.1|odorant receptor 140 [*Nasonia vitripennis*]	7E–26	39	0.65	0.36
McinOR61	c19425_g2	837	279	NP_001177600.1|odorant receptor 255 [*Nasonia vitripennis*]	5E–31	30	0.57	0.86
McinOR62	c19470_g1	940	287	AIG51906.1|odorant receptor [*Helicoverpa armigera*]	2E–41	31	0.89	N/A
McinOR63	c19502_g2	626	287	AID59306.1|odorant receptor 8 [*Macrocentrus cingulum*]	1E–138	96	0.67	0.68
McinOR64	c19559_g1	672	192	NP_001177594.1|odorant receptor 245 [*Nasonia vitripennis*]	1E–29	30	0.49	N/A
McinOR65	c19682_g1	935	307	NP_001164418.1|odorant receptor 246 [*Nasonia vitripennis*]	1E–51	32	0.55	N/A
McinOR66	c19687_g1	1125	342	XP_011315403.1|PREDICTED: odorant receptor 67a-like [Fopius arisanus]	2E–78	37	0.41	N/A
McinOR67	c19759_g1	1075	286	NP_001177702.1|odorant receptor 156 [Nasonia vitripennis]	1E–45	27	0.29	0.53
McinOR68	c19759_g3	1229	244	NP_001177576.1|odorant receptor 204 [Nasonia vitripennis]	2E–34	24	2.89	0.62
McinOR69	c19813_g1	640	171	NP_001177596.1|odorant receptor 248 [Nasonia vitripennis]	3E–32	32	0.25	0.67
McinOR70	c19813_g3	1040	317	NP_001177602.1|odorant receptor 257 [*Nasonia vitripennis*]	6E–51	31	0.32	0.75
McinOR71	c19862_g1	459	107	NP_001164460.2|odorant receptor 262 [*Nasonia vitripennis*]	2E–13	27	0.53	0.82
McinOR72	c19862_g2	793	230	NP_001164417.1|odorant receptor 243 [*Nasonia vitripennis*]	2E–18	22	0.39	0.76
McinOR73	c19954_g1	717	130	NP_001229918.1|odorant receptor 115 [*Apis mellifera*]	5E–51	38	0.71	0.84
McinOR74	c19972_g1	1173	391	NP_001177544.1|odorant receptor 142 [*Nasonia vitripennis*]	2E–59	29	0.14	0.69
McinOR75	c20051_g3	418	132	XP_011262032.1|PREDICTED: odorant receptor 30a-like [Camponotus floridanus]	2E–20	36	0.59	1.09
McinOR76	c20052_g1	1137	379	XP_011250449.1|PREDICTED: odorant receptor Or2–like [*Camponotus floridanus*]	5E–51	32	0.38	0.96
McinOR77	c20066_g1	659	206	AGG17937.1|olfactory receptor 4 [*Microplitis mediator*]	3E–30	32	1.04	1.32
McinOR78	c20071_g2	1092	297	AGG17942.1|olfactory receptor 10 [*Microplitis mediator*]	5E–54	33	0.70	0.99
McinOR79	c20071_g3	804	267	XP_011644881.1|PREDICTED: odorant receptor 4-like [*Pogonomyrmex barbatus*]	5E–59	37	0.75	0.89
McinOR80	c20071_g4	634	202	XP_011645081.1|PREDICTED: odorant receptor 85f-like [*Pogonomyrmex barbatus*]	7E–25	28	1.12	0.90
McinOR81	c20107_g1	1171	390	NP_001164399.1|odorant receptor 92 [*Nasonia vitripennis*]	4E–46	28	0.40	1.01
McinOR82	c20110_g1	1170	390	NP_001177423.1|odorant receptor 159 [*Nasonia vitripennis*]	2E–41	26	0.18	0.94
McinOR83	c20171_g1	856	257	AHJ37466.1|olfactory receptor 152 isoform 1 [*Apis mellifera*]	1E–23	28	0.62	0.94
McinOR84	c22092_g1	501	167	NP_001164419.1|odorant receptor 249 [*Nasonia vitripennis*]	3E–06	22	2.51	1.00
McinOR85	c24180_g1	1117	341	XP_011311726.1|PREDICTED: odorant receptor 46a, isoform A-like isoform X2 [*Fopius arisanus*]	6E–45	30	0.39	0.92
McinOR86	c31215_g1	1083	336	NP_001177621.1|odorant receptor 292 [*Nasonia vitripennis*]	1E–12	23	0.32	1.02
McinOR87	c37356_g1	420	138	NP_001177595.1|odorant receptor 247 [Nasonia vitripennis]	8E–15	27	2.51	1.30
McinOR88	c37828_g1	502	164	XP_012542767.1|PREDICTED: putative odorant receptor 92a [*Monomorium pharaonis*]	5E–05	22	N/A	0.88

Comparative gene expression is reported as a ratio of female to male (F:M) transcript levels estimated by depth among RNA-seq reads (RSq) and RT-PCR.

**Table 5:** Candidate gustatory receptor genes identified in adult male and female *M. cingulum* antennal transcriptomes.

Gene Name	Contig ID	Contig (bp)	ORF (aa)	BLASTx best hit (GenBank acsession/name/species)	E- value	% ID	F:M
							RSq
McinGR1	c8912_g1	945	237	AKC58582.1|gustatory receptor 5 [*Anomala corpulenta*]	1E–105	57	1.02
McinGR2	c10432_g1	745	248	XP_008551044.1|PREDICTED: putative gustatory receptor 28b [*Microplitis demolitor*]	1E–25	31	2.18
McinGR3	c11295_g1	829	262	NP_001177445.1|gustatory receptor 25 [*Nasonia vitripennis*]	2E–10	27	0.41
McinGR4	c11371_g1	410	127	NP_001177460.1|gustatory receptor 43 [*Nasonia vitripennis*]	2E–08	26	0.30
McinGR5	c11414_g2	881	288	NP_001177436.1| gustatory receptor 10 [*Nasonia vitripennis*]	4E–39	34	0.76
McinGR6	c14786_g1	1239	413	NP_001177449.1| gustatory receptor 30 [*Nasonia vitripennis*]	8E–24	25	0.33
McinGR7	c14813_g1	913	304	|NP_001177460.1| gustatory receptor 43 [*Nasonia vitripennis*]	0.11	21	0.35
McinGR8	c14834_g1	1386	400	NP_001164385.1| gustatory receptor 2 [*Nasonia vitripennis*]	2E–104	51	1.04
McinGR9	c16638_g1	1209	377	NP_001177441.1| gustatory receptor 15 [*Nasonia vitripennis*]	5E–08	48	0.96
McinGR10	c16671_g1	1203	401	NP_001177444.1| gustatory receptor 22 [*Nasonia vitripennis*]	1E–04	21	0.65
McinGR11	c17466_g1	708	212	NP_001177447.1| gustatory receptor 28 [*Nasonia vitripennis*]	0.081	21	0.94
McinGR12	c17727_g1	705	217	NP_001177460.1| gustatory receptor 43 [*Nasonia vitripennis*]	4E–18	28	0.22
McinGR13	c19250_g1	834	255	AKC58578.1| gustatory receptor 3, partial [*Anomala corpulenta*]	7E–09	21	0.47
McinGR14	c19552_g1	1362	448	AKO90019.1| gustatory receptor 6 [*Microplitis mediator*]	4E–128	50	0.73
McinGR15	c19791_g1	1191	353	AKO90018.1|gustatory receptor 64f [*Microplitis mediator*]	2E–93	39	0.52
McinGR16	c19791_g2	1347	449	P_003708225.1|PREDICTED: putative gustatory receptor 28a isoform X1 [*Megachile rotundata*]	2E–62	31	0.96
McinGR17	c19791_g3	459	142	|XP_003708225.1| PREDICTED: putative gustatory receptor 28a isoform X1 [*Megachile rotundata*]	2E–19	36	0.36
McinGR18	c27251_g1	597	198	NP_001177453.1|gustatory receptor 35 [*Nasonia vitripennis*]	5E–11	31	0.24
McinGR19	c28135_g1	315	79	EFA07633.1| gustatory receptor 155 [*Tribolium castaneum*]	4E–08	32	0.60
McinGR20	c31991_g1	416	138	NP_001164388.1|gustatory receptor 47 [*Nasonia vitripennis*]	0.019	21	NPF

NPF = not present in female libraries (exclusive male expression).

**Table 6:** Candidate ionotropic receptor genes identified in adult male and female *M. cingulum* antennal transcriptomes.

Gene Name	Contig ID	Contig (bp)	ORF (aa)	BLASTx best hit (GenBank acsession/name/species)	E– value	% ID	F:M
							RSq
McinIR8a	c14535_g1	2724	908	KO90022.1|ionotropic receptor 8a [*Microplitis mediator*]	0.0	75	0.26
McinIR64a.1	c15376_g1	2064	688	AKO90024.1| ionotropic receptor 64a [*Microplitis mediator*]	0.0	44	0.12
McinIR76b	c16560_g1	1578	472	AKO90021.1| ionotropic receptor 76b [*Microplitis mediator*]	1E–150	46	0.75
McinIR7e.1	c16617_g1	1533	511	XP_008547712.1|PREDICTED: glutamate receptor ionotropic, kainate 2 isoform X3 [*Microplitis demolitor*]	0.0	90	1.16
McinIR64a.2	c16617_g2	1467	489	XP_011303607.1|PREDICTED: glutamate receptor ionotropic, kainate 2 isoform X4 [*Fopius arisanus*]	0.0	88	1.26
McinIR7e.2	c17848_g2	2529	743	XP_011304727.1|PREDICTED: glutamate receptor ionotropic, kainate 2 isoform X1 [*Fopius arisanus*]	0.0	87	2.19
McinIR25a.1	c18109_g4	2865	916	AKO90023.1|ionotropic receptor 25a.1 [*Microplitis mediator*]	0.0	57	1.01
McinIR63a.3	c18122_g1	1783	394	BAR64801.1| ionotropic receptor [*Ostrinia furnacalis*]	4E–84	31	0.15
McinIR7e.3	c18998_g1	2035	633	XP_012344615.1| PREDICTED: glutamate receptor ionotropic, kainate 2-like isoform X2 [*Apis florea*]	0.0	58	1.66
McinIR7e.4	c18998_g2	1722	513	AIG51927.1| ionotropic glutamate receptor [*Helicoverpa armigera*]	4E–154	50	0.90
McinIR75u	c19512_g1	1557	518	AKO90020.1|ionotropic receptor 75u [*Microplitis mediator*]	0.0	61	0.48
McinIR21a	c19661_g2	1065	326	AJO62240.1|chemosensory ionotropic receptor IR2 [*Tenebrio molitor*]	6E–18	23	1.26
McinIR93a	c20064_g1	2643	881	NP_650924.3| ionotropic receptor 93a [*Drosophila melanogaster*]	6E–128	32	0.50
