# Supplementary material for: Gene set of chemosensory receptors in the polyembryonic endoparasitoid Macrocentrus cingulum
Source: Sci Rep. 2016 Apr 19;6:24078. doi: 10.1038/srep24078 (PMC4835793; doi:10.1038/srep24078)
Supplement: Supplementary Information [file srep24078-s1.pdf]

Gene set of chemosensory receptors in the polyembryonic endoparasitoid *Macrocentrus cingulum*

Tofael Ahmed[1, 2], Tiantao Zhang[1*], Zhenying Wang[1*], Kanglai He[1], Shuxiong Bai[1]

1.State Key Laboratory for the Biology of the Plant Diseases and Insect Pests, Institute of Plant Protection, Chinese Academy of Agricultural Sciences, Beijing 100193, China;

2. Bangladesh Sugar Crops Research Institute, Ishurdi-6620, Pabna, Bangladesh;

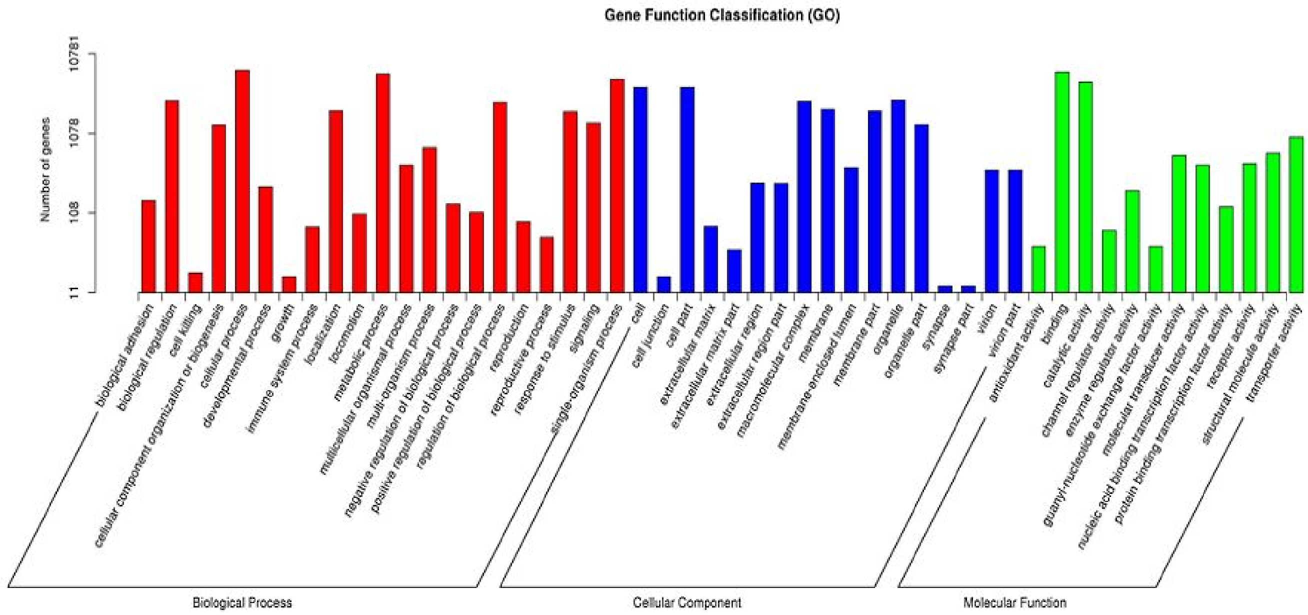

Figure S1 **Gene ontology level 2 results.** Gene ontology analyses as in Figure 2, but here represented as bar diagrams that have a higher resolution.

Table S1. Primer used in RT-PCR.

Gene No.	Contig No.	Primer	Sequence (5' to 3')
McinOR10	c592_g1	c592_g1 F	CAGGAGGATGGTTGATTATTTG
		c592_g1 R	GAGGATAAGGGCGTTAGATATTG
McinOR11	c1234_g1	c1234_g1 F	GATGGCGCTTACAAAAGTATG
		c1234_g1 R	GTAAAGCTTCAATCGTTGCTG
McinOR12	c2517_g1	c2517_g1 F	CCTAGTTATATGGGTTATTGCACTG
		c2517_g1 R	GAAGTTTAGCCACAATTCC
McinOR13	c4077_g1	c4077_g1 F	ATGCCAGACAAACAACTGAG
		c4077_g1R	CATAAATGATGGTAGAGTCAGATC
McinOR14	c8354_g1	c8354_g1 F	CGGATTTACCATGAAATACAGAC
		c8354_g1 R	GAAATCTCATGCTGCAGAACAG
McinOR15	c9201_g1	c9201_g1F	CAAATGATGGGTAGGCTGC
		c9201_g1 R	CGCTGGGGAAAATCTTACTG
McinOR16	c9490_g1	c9490_g1 F	GATCCCACCCAATGGATTTCTTTAC
		c9490_g1 R	GCTGGAGAGTTTGAAGTGCAAAC
McinOR17	c9743_g1	c9743_g1 F	GAATAATGGCCTACGGTTGC
		c9743_g1 R	GTACCAGCATAGCACACGAC
McinOR18	c11069_g1	c11069_g1 F	GTACTCTGCCCTGACCTTTTC
		c11069_g1 R	GATGTTTCGGAGCATCTGC
McinOR19	c11500_g1	c11500_g1 F	ATGTCGAAGAAGAATTTATCAGAC
		c11500_g1 R	GTAAAGCATCCCATTGGACTC
McinOR20	c11576_g1	c11576_g1 F	CATGGAGAGAGATTGGCTTACTAC
		c11576_g1 R	GATTGAAAGAGTGCCAACAGC
McinOR21	c11829_g1	c11829_g1 F	GTTGATGCATCAGCAATTGTG
		c11829_g1 R	GATTATCCCACCGAAATTCTG
McinOR22	c11862_g1	c11862_g1 F	GTACATTGGCTTGGTCTCAAG
		c11862_g1 R	GTCAAGTGCTGACCAATAAAAG
McinOR23	c12194_g1	c12194_g1 F	CTCGGTATTTGGCCTCTTG
		c12194_g1 R	CATACGTGCATTGCGATC
McinOR24	c12240_g1	c12240_g1 F	CTTCAGATTGCCAGCATTC
		c12240_g1 R	CTTTCCAGCAGTCAGTTGC
McinOR25	c12488_g1	c12488_g1 F	GTATGGACGACAAATCCGC
		c12488_g1 R	CCACAGAGAACAATAACTTGCG
McinOR26	c12943_g1	c12943_g1 F	ATCAAAGAGTGTTTCGAGCACATG
		c12943_g1 R	CTCAAAACATTTAAATAAATTACCGATG
McinOR27	c12996_g1	c12996_g1 F	CATGATATTTCGCGAGTCTAATC
		c12996_g1 R	CTGCAAATTCCTGTCATAACAC
McinOR28	c13179_g1 (2)	c13179_g1 F(2)	GTGTTTGCTACACAAGCTCTC
		c13179_g1 R(2)	CATTTTGGCTGCTCTGC
McinOR29	c13458_g1	c13458_g1 F	CATGGTGAATCGGAAAGATATC
		c13458_g1 R	CGAAATAGCAGTGTTTGTGACC
McinOR30	c13476_g1	c13476_g1 F	ATGAAGATAAGCGTCGATTCAAC

		c13476_g1 R	GAAACTTGTTCGAAAGAATGTCC
McinOR31	c13904_g1	c13904_g1 F	CTTCTCCACTATGTACGGC
		c13904_g1 R	CTGAAGCAAGCACCATTAAG
McinOR32	c14017_g1	c14017_g1 F	GTGCCAATTACTTGGAATATGG
		c14017_g1 R	GATAGGCATGAAGTGTAATTGGTAC
McinOR33	c14398_g1	c14398_g1 F	CACCATGTACAGAACAATGAGTC
		c14398_g1 R	GAAGTGCGTGATTGTGTTTC
McinOR34	c14712_g1	c14712_g1 F	GATGGCGATATTATCCGAAC
		c14712_g1 R	CCAGTAGCCATAATACTCATAATCG
McinOR35	c15245_g1	c15245_g1 F	CTTGGATTATGGCTATATCCTAACG
		c15245_g1 R	GAATTTTCAATTGGCCGC
McinOR36	c15542_g1	c15542_g1 F	CAGATGTTTTCTGAGATGACG
		c15542_g1 R	GAAAGAGTGCATCAACTCCAC
McinOR37	c15710_g1	c15710_g1 F	CATGGTGCAAATGGTTATGAG
		c15710_g1 R	CTTTAAATAGCTTGGCGTAGAACTG
McinOR38	c15806_g1	c15806_g1 F	CAATGTTTTCATTCACTCTGC
		c15806_g1 R	CCGCCAGATCTTTCAAGC
McinOR39	c15883_g1	c15883_g1 F	GTCACTCAGAGTCAGAAGAATTCTG
		c15883_g1 R	CGTTATTGCATAGATCTCAAAACC
McinOR40	c15902_g1	c15902_g1 F	ATGAACCGTGAGAATGTGTC
		c15902_g1 R	CATTACCCACAAAATCATCG
McinOR41	c15957_g1	c15957_g1 F	GTATGATTGCTGCCTTCTCG
		c15957_g1 R	GATTCGCGCAAATTCTATTG
McinOR42	c16612_g1	c16612_g1 F	CATGGAATACGAGAAACTGATAG
		c16612_g1 R	GTTCAGCAAAGAAAATTGCATC
McinOR43	c16640_g1	c16640_g1 F	GATGATAGCAGCTTGGTGTG
		c16640_g1 R	GTGCATATTCCAAAGTCTGACTG
McinOR44	c16673_g1	c16673_g1 F	GTTATGTATATTGAGGTCTATCGAG
		c16673_g1 R	GCTACGTAACACCGAGAGATAC
McinOR45	c16974_g2	c16974_g2 F	CAATGGACGGGAATAGTATGAC
		c16974_g2 R	CACAAACATGCATAGCAATGC
McinOR46	c16982_g1	c16982_g1 F	CAAGCAATGTTGAGGTGGAG
		c16982_g1 R	CTTATTGTATTGAGGTGATTGATGC
McinOR47	c17113_g1	c17113_g1 F	GAAAATGTGGAGCACAATTG
		c17113_g1 R	GCGACAAGTGTTGTCTCGAC
McinOR48	c17665_g1	c17665_g1 F	GCTTAGCAATCGAAAACCAC
		c17665_g1 R	CGATGCATGTAACGAAGCTC
McinOR49	c17700_g1	c17700_g1 F	CATGATTCATCATCAATCAATTTTAG
		c17700_g1 R	CCGAGAGTTGGCGGAGTAC
McinOR50	c17768_g1	c17768_g1 F	CATGTGGACAACATTCAACGAC
		c17768_g1 R	GCCGCAAGTAAGTGCATAAC
McinOR51	c17907_g1	c17907_g1 F	GAATGATGTTTTTGGTCTGTGC
		c17907_g1 R	GTTGAATGACATTGTAAATCGAG
McinOR52	c18012_g1	c18012_g1 F	ATGGAGACCGCAGAAATC

		c18012_g1 R	CTGTACTGAAGTATTGCAATTTGC
McinOR53	c18194_g2	c18194_g2 F	CAGTTTGAAATTCTGAAAAGCAG
		c18194_g2 R	GGATTGTTTTAGAACGTTGTAAGC
McinOR54	c18310_g1	c18310_g1 F	ATGAGTACAATGCAGCGACG
		c18310_g1 R	GTCAAAGAAAGCACGAGACAC
McinOR55	c18322_g1	c18322_g1 F	GATCCATTTGATGACGGTCAC
		c18322_g1 R	CTGAAGAAAGTACCATTAAATAAGACATTG
McinOR56	c18374_g1	c18374_g1 F	GTATTGTAGTTTGCAGCAAGC
		c18374_g1 R	GATCCCTCCTACAGAGATGAGTC
McinOR57	c18647_g1	c18647_g1 F	ATGAAGATTCTCACGGAACAC
		c18647_g1 R	CAGTTGTCCACAAATATGTATCAGC
McinOR58	c18685_g1	c18685_g1 F	CGAAAAACATCAATTGATAGTAACTG
		c18685_g1 R	CAAATCTGCGCGATAGACAC
McinOR59	c18814_g2	c18814_g2 F	CATGACCGATGAGATAGAGGC
		c18814_g2 R	CACTTTATTTATTGCTGTGCAGAG
McinOR60	c19087_g1	c19087_g1 F	GAGTCATCAAGAACTAGTTCAATG
		c19087_g1 R	GAAATGATAACAAAACAGTCAAG
McinOR61	c19425_g2	c19425_g2 F	CAATGTACTTGGCATATCGC
		c19425_g2 R	CACGAAATTGTCCCATCATC
McinOR62	c19470_g1	c19470_g1 F	GTCTACAGCTTGGGAACCTTC
		c19470_g1 R	CAAGAGTTGGAGATTGCGC
McinOR63	c19502_g2	c19502_g2 F	GAGCGAGATGTAATGTTAAAATACG
		c19502_g2 R	CCAGTAGAAAAGATATATAGCTTCCTG
McinOR64	c19559_g1	c19559_g1 F	GACAATCTGCATAATGGCAAATC
		c19559_g1 R	CTCTTCGTCAATAGTTTGAAGCAGG
McinOR65	c19682_g1	c19682_g1 F	GATTATGTGCATTGCATTCAACTAC
		c19682_g1 R	GACATGTTCGGCAGTTAAATG
McinOR66	c19687_g1	c19687_g1 F	GTCAGAGCTTATTGAGCTGCTG
		c19687_g1 R	CGACAAACCATAGATGCTAACAC
McinOR67	c19759_g1	c19759_g1 F	GAATAACTTGATCGCAATAACGATG
		c19759_g1 R	GATTCTTGAACAGATCTTAAAAGAGTG
McinOR68	c19759_g3	c19759_g3 F	ATGAGTATCCTGGATCACCCTC
		c19759_g3 R	CCGAACGTAAGAGGGTAAAATAC
McinOR69	c19813_g1	c19813_g1 F	GATTGCTCAACTTCGAGAAATG
		c19813_g1 R	CCTTATCTTGAAGCGTTTGTAAGAG
McinOR70	c19813_g3	c19813_g3 F	AAATTATCAGAATACCTGATATTCCGG
		c19813_g3 R	GATGCTGCATGGCTTCTCTTC
McinOR71	c19862_g1	c19862_g1 F	CACAAATGATTGGCCAGTTTATAC
		c19862_g1 R	CAAGTGCGTAGAGAAACGTTTG
McinOR72	c19862_g2	c19862_g2 F	CAATGACTTTCTTTATTCGCAAG
		c19862_g2 R	CAAATATTTCCAGCATTTGTCAG
McinOR73	c19954_g1	c19954_g1 F	GATCTCATACAAGTCTTGTCTAGCC
		c19954_g1 R	GATAATCGATTTTCAACAGTCAGC
McinOR74	c19972_g1	c19972_g1 F	CTTACTGTTCTTGGGCTATGG

		c19972_g1 R	CTATTTTTTCGTAGATCAACTGCG
McinOR75	c20051_g3	c20051_g3 F	GATATAAATCGTGCATCTGTTGG
		c20051_g3 R	GAATTCACGGGAATTTTCAAC
McinOR76	c20052_g1	c20052_g1 F	GATTAGCGACATACGGTTTTG
		c20052_g1 R	CAGTTGATTCCACTGAAGATGAC
McinOR77	c20066_g1	c20066_g1 F	CAGTACGCTGATTATTTACACGC
		c20066_g1 R	GATGCTGATGTCTTCAGAATCG
McinOR78	c20071_g2	c20071_g2 F	GCTATGTGTGCTTTAAAATTTTGTG
		c20071_g2 R	GCAATTTCTCGACGCAGAC
McinOR79	c20071_g3	c20071_g3 F	CTCAGAAAACTCTTGCCAGC
		c20071_g3 R	CCAAGTTGTCCACAAATATAACTC
McinOR80	c20071_g4	c20071_g4 F	ATGCCGCGCAGATTAAC
		c20071_g4 R	CAGTGATGGCTGATGATAGTAGC
McinOR81	c20107_g1	c20107_g1 F	GTTTATGATGCTGTCAATGGC
		c20107_g1 R	GCACGAGTAGAGCTGATATGTAAAC
McinOR82	c20110_g1	c20110_g1 F	GAGTAATAGGTAGATGGCCAAATC
		c20110_g1 R	GTATTCGATTACTGATTGATGTCC
McinOR83	c20171_g1	c20171_g1 F	GTGGTGCCTAATGTTGATAATTTC
		c20171_g1 R	CTTCTTAAAACTTCCGATGGC
McinOR84	c22092_g1	c22092_g1 F	ATGAGCTTTGTAACAGTCTTTGTC
		c22092_g1 R	GAATGACAATCCTAGTGTCGC
McinOR85	c24180_g1	c24180_g1 F	GATCACAGATATTCGATTACTTTCG
		c24180_g1 R	GTAACGAGCATGGCAGTATGAG
McinOR86	c31215_g1	c31215_g1 F	CTTTATGGTCTACCTTGATTTGATTGC
		c31215_g1 R	GTTGACAGTTCGTAGCATTGATG
McinOR87	c37356_g1	c37356_g1 F	CTCATGCAATGCCGTGATCTTC
		c37356_g1 R	ATCGTTTTGAATCGCTCGCAG
McinOR88	c37828_g1	c37828_g1 F	CGTTTGTTATGCTAAGACTCGC
		c37828_g1 R	CCAAAATCCAAGACCTGAGTG
McinGR1	c8912_g1	c8912_g1 F	GGCTAAGAAGACACGCAACA
		c8912_g1 R	CAGCGAAACTGCCACATTAC
McinGR2	c10432_g1	c10432_g1 F	CAGGGTTTTAGGGTGGAAAA
		c10432_g1 R	AGGCTGAGAGGACGAAATACA
McinGR3	c11295_g1	c11295_g1 F	GTCCAATTGGGCGGTTATA
		c11295_g1 R	CGTCGAATCGAGTTGAGATG
McinGR4	c11371_g1	c11371_g1 F	CCCGATCGTTACGCTAAGTT
		c11371_g1 R	TCCACAAGCAGTGAATGACA
McinGR5	c11414_g2	c11414_g2 F	ATGCTCTTCCTCACCACCAT
		c11414_g2 R	CGCACAAATACGTCAAAACC
McinGR6	c14786_g1	c14786_g1 F	TGTTCTCCGTACGTCAATCG
		c14786_g1 R	CGGCTTCAGCAGTAGTCGTA
McinGR7	c14813_g1	c14813_g1 F	CAAGTGAATCGTCCGAAAAA
		c14813_g1 R	GCGAAGGGTACGTAGATCGT

McinGR8	c14834_g1	c14834_g1 F	CTCGAGTTTTTGGTCTGCTG
		c14834_g1 R	ATCGAATGAATCGGTGTTGA
McinGR9	c16638_g1	c16638_g1 F	CCGATTCATTGAACCAAATG
		c16638_g1 R	TTTCCGGTGCAATAAAGTGA
McinGR10	c16671_g1	c16671_g1 F	CTGGCTTCTCACTGCGATTA
		c16671_g1 R	GCAAAGGCCTCGTTAATACTG
McinGR11	c17466_g1	c17466_g1-F	AGTGCACAGTTGACAGTTTT
		c17466_g1-R	GTGTGCCCTCAACTTGGAGG
McinGR12	c17727_g1	c17727_g1 F	CAACCAAATGTTCGATTGGA
		c17727_g1 R	TAAATGAACGGTCGAGCAAG
McinGR13	c19250_g1	c19250_g1 F	AAACCAGGCTTTTGGCATAG
		c19250_g1 R	GGCCTGGGTAACGTACAAAA
McinGR14	c19552_g1	c19552_g1 F	TACTTTGCCGTCTCGTTGAC
		c19552_g1 R	GAAAACACCAGAGTCGCTGA
McinGR15	c19791_g1	c19791_g1 F	AGGATAGCGAGGATCTGGAA
		c19791_g1 R	CACGTCTCGTTAAAGGCGTA
McinGR16	c19791_g2	c19791_g2 F	CAAATCCAGAGCTACGACGA
		c19791_g2 R	TAGGCCACATTTCTGAACCA
McinGR17	c19791_g3	c19791_g3 F	CTGAGCGACCTGTACTCCTG
		c19791_g3 R	AAGAATGAAGAGCTGCACGA
McinGR18	c27251_g1	c27251_g1 F	GGGTTTTTGCTTGGGAAATA
		c27251_g1 R	TGTCAATTTGAGTGACTGTGTG
McinGR19	c28135_g1	c28135_g1 F	TGCTGCAAATGACACACAAT
		c28135_g1 R	TTCGCGAGTCAAGTTGAAAT
McinGR20	c31991_g1	c31991_g1 F	TGCACAGAATTTTACGCACA
		c31991_g1 R	GGAAGAAATGCCGTGAAGAT
McinIR8a	c14535_g1	c14535_g1 F	TCCAGCCACAGACAACTGTA
		c14535_g1 R	CATCAATGTCGTCTCGATCA
McinIR64a.1	c15376_g1	c15376_g1 F	TGGGCTGAGAAAAATCGTAG
		c15376_g1 R	AATGCCAGTCCCATGAGTAA
McinIR76b	c16560_g1	c16560_g1 F	AATTTTGCTCGTACGGTGTC
		c16560_g1 R	GGTGATGACGAAGAAAATCG
McinIR7e.1	c16617_g1	c16617_g1 F	CAGTGATCTCAATCCCAAGG
		c16617_g1 R	TGTCAGTTTGTCCAGCAAGA
McinIR64a.2	c16617_g2	c16617_g2 F	GGGGTAAACATCACAGATGC
		c16617_g2 R	TTCTTTTAGCAGGTCGATGC
McinIR7e.2	c17848_g2	c17848_g2 F	TGGGCTGAAGAAAATTGGTA
		c17848_g2 R	TCAGCTCCATCGTAGAGTCC
McinIR25a.1	c18109_g4	c18109_g4 F	AAGGAGGAGACTGCGAAAAT
		c18109_g4 R	GTAAGCTCGTCGTTCATCGT
McinIR63a.3	c18122_g1	c18122_g1 F	GTTATCGTCATGGAGGCAAG
		c18122_g1 R	ATTGTAGTCCGGAACTGCTG
McinIR7e.3	c18998_g1	c18998_g1 F	CTATAGGAGCGTGGAACAGC
		c18998_g1 R	TCATTTCCAACCTTCATCGT

McinIR7e.4	c18998_g2	c18998_g2 F	TTACCACCAAACCTGGAGAA
		c18998_g2 R	TACTGCTTGTCGGGAACAAT
McinIR75u	c19512_g1	c19512_g1 F	GATTTTACGTGGCGCAATAC
		c19512_g1 R	GAACCAACGGGTGTGTAGAG
McinIR21a	c19661_g2	c19661_g2 F	ACGAATTTATGCGCAATACC
		c19661_g2 R	AGTCGTTTTCAGGGCTCTTC
McinIR93a	c20064_g1	c20064_g1 F	CTTGTGAGGAGCAGACACCT
		c20064_g1 R	TGGCTTCGAATTTGACTCTC
Actin	*β-actin*	Mcinactin F	CATGGAGAAGATTTGGCACC
		Mcinactin R	CGATAGTGATGACCTGTCCGTCG

Table S2. Candidate odorant receptor transcripts (≤100 aa) identified in adult male and female *Macrocentrus cingulum* antennal transcriptomes

**Transcript Name**	**Contig (bp)**	**ORF (aa)**	**BLASTx best hit (GenBank acsession/name/species)**	**E value**	**Similarity (%)**
c1290_g1	319	83	XP_011334210.1| odorant receptor 45b-like [*Cerapachys biroi*]	6E-10	34
c2292_g1	216	59	NP_001177575.1| odorant receptor 203 *[Nasonia vitripennis]*	5E-04	28
c3162_g1	350	87	NP_001177604.1|odorant receptor 261 [*Nasonia vitripennis*]	2E-13	32
c3821_g1	398	58	NP_001164394.1| odorant receptor 81 [*Nasonia vitripennis*]	4E-13	31
c7096_g1	314	69	|XP_011135383.1|odorant receptor 43a-like [*Harpegnathos saltator*]	3E-16	42
c9531_g1	314	62	NP_001229906.1| odorant receptor 52 [*Apis mellifera*]	2E-13	36
c9975_g1	311	95	NP_001177541.1| odorant receptor 137 [*Nasonia vitripennis*]	2E-18	40
c12179_g1	385	70	AGG17940.1| olfactory receptor 7 [*Microplitis mediator*]	1E-26	38
c14258_g1	206	49	XP_008561014.1| PREDICTED: odorant receptor Or2-like [*Microplitis demolitor*]	6E-06	45
c17369_g1	394	77	AGG17939.1| olfactory receptor 6 [*Microplitis mediator*]	1E-18	44
c18797_g1	362	93	NP_001164404.1|odorant receptor 141 [*Nasonia vitripennis*]	1E-18	36
c19425_g4	298	81	NP_001177709.1|odorant receptor 258 [*Nasonia vitripennis*]	2E-18	44
c21485_g1	260	77	NP_001229895.1|odorant receptor 19 [*Apis mellifera*]	5E-09	36
c24037_g1	361	51	|NP_001164671.1|odorant receptor 77 [*Nasonia vitripennis*]	0.001	27
c25757_g1	243	72	NP_001229905.1|odorant receptor 51 [*Apis mellifera*]	4E-07	30
c26191_g1	283	31	XP_012534748.1|PREDICTED: odorant receptor 4-like isoform X2 [*Monomorium pharaonis*]	2E-08	34
c30579_g1	218	54	NP_001177597.1|odorant receptor 250 [*Nasonia*	8E-03	30

			vitripennis]		
c31910_g1	182	48	AIT71989.1|olfactory receptor 18 [*Ctenopseustis obliquana*]	0.83	33
c34725_g1	222	45	NP_001177598.1|odorant receptor 251 [*Nasonia vitripennis*]	1E-13	49
c37164_g1	337	61	NP_001229900.1|odorant receptor 35 [*Apis mellifera*]	9E-10	33

Table S3. Primer used in qPCR.

Gene No.	Contig No.	Primer	Sequence (5' to 3')
McinGR1	c8912_g1	c8912_g1 F	GGCTAAGAAGACACGCAACA
		c8912_g1 R	CAGCGAAACTGCCACATTAC
McinGR2	c10432_g1	c10432_g1 F	CAGGGTTTTAGGGTGGAAAA
		c10432_g1 R	AGGCTGAGAGGACGAAATACA
McinGR3	c11295_g1	c11295_g1 F	GTCCAATTGGGCGGTTATA
		c11295_g1 R	CGTCGAATCGAGTTGAGATG
McinGR4	c11371_g1	c11371_g1 F	CCCGATCGTTACGCTAAGTT
		c11371_g1 R	TCCACAAGCAGTGAATGACA
McinGR5	c11414_g2	c11414_g2 F	ATGCTCTTCCTCACCACCAT
		c11414_g2 R	CGCACAAATACGTCAAAACC
McinGR6	c14786_g1	c14786_g1 F	TGTTCTCCGTACGTCAATCG
		c14786_g1 R	CGGCTTCAGCAGTAGTCGTA
McinGR7	c14813_g1	c14813_g1 F	CAAGTGAATCGTCCGAAAAA
		c14813_g1 R	GCGAAGGGTACGTAGATCGT
McinGR8	c14834_g1	c14834_g1 F	CTCGAGTTTTTGGTCTGCTG
		c14834_g1 R	ATCGAATGAATCGGTGTTGA
McinGR9	c16638_g1	c16638_g1 F	CCGATTCATTGAACCAAATG
		c16638_g1 R	TTTCCGGTGCAATAAAGTGA
McinGR10	c16671_g1	c16671_g1 F	CTGGCTTCTCACTGCGATTA
		c16671_g1 R	GCAAAGGCCTCGTTAATACTG
McinGR11	c17466_g1	c17466_g1-F	AGTGCACAGTTGACAGTTTT
		c17466_g1-R	GTGTGCCCTCAACTTGGAGG
McinGR12	c17727_g1	c17727_g1 F	CAACCAAATGTTCGATTGGA
		c17727_g1 R	TAAATGAACGGTCGAGCAAG
McinGR13	c19250_g1	c19250_g1 F	AAACCAGGCTTTTGGCATAG
		c19250_g1 R	GGCCTGGGTAACGTACAAAA
McinGR14	c19552_g1	c19552_g1 F	TACTTTGCCGTCTCGTTGAC
		c19552_g1 R	GAAAACACCAGAGTCGCTGA
McinGR15	c19791_g1	c19791_g1 F	AGGATAGCGAGGATCTGGAA
		c19791_g1 R	CACGTCTCGTTAAAGGCGTA
McinGR16	c19791_g2	c19791_g2 F	CAAATCCAGAGCTACGACGA
		c19791_g2 R	TAGGCCACATTTCTGAACCA

McinGR17	c19791_g3	c19791_g3 F	CTGAGCGACCTGTACTCCTG
		c19791_g3 R	AAGAATGAAGAGCTGCACGA
McinGR18	c27251_g1	c27251_g1 F	GGGTTTTTGCTTGGGAAATA
		c27251_g1 R	TGTCAATTTGAGTGACTGTGTG
McinGR19	c28135_g1	c28135_g1 F	TGCTGCAAATGACACACAAT
		c28135_g1 R	TTCGCGAGTCAAGTTGAAAT
McinGR20	c31991_g1	c31991_g1 F	TGCACAGAATTTTACGCACA
		c31991_g1 R	GGAAGAAATGCCGTGAAGAT
